# Supplementary material for: UpSetR: an R package for the visualization of intersecting sets and their properties
Source: Bioinformatics. 2017 Jun 22;33(18):2938–40. doi: 10.1093/bioinformatics/btx364 (PMC5870712; doi:10.1093/bioinformatics/btx364)
Supplement: Supplementary Data [file btx364_supplementary.pdf]

Supplementary Material

UpSetR: An R Package for the Visualization of Intersecting Sets and their Properties

Jake R Conway[1], Alexander Lex[2], and Nils Gehlenborg[1*]

[1]Department of Biomedical Informatics, Harvard Medical School, Boston, MA 02115, USA
[2]SCI Institute, School of Computing, University of Utah, Salt Lake City, UT 84112, USA
[*]Corresponding Author: nils@hms.harvard.edu

Supplementary Information

The data used in the usage scenario was retrieved from the Data Coordination Center (DCC) of the International Cancer Genomics Consortium (https://dcc.icgc.org/) on March 18, 2016 through the `projects` API endpoint using an R script that also converted the data into a format readable by UpSetR. All code used to retrieve the data, a copy of the data used for Figures 1 and Supplementary Figures 3 through 5, as well as the code used to generate said figures, is available on GitHub: https://github.com/hms-dbmi/UpSetR-paper.

We retrieved data for the following eight ICGC cancer cohorts: LUSC-CN (Lung Squamous Cell Carcinoma, China), LUSC-KR (Lung Squamous Cell Carcinoma, Korea), LUSC-US (Lung Squamous Cell Carcinoma, USA), THCA-SA (Thyroid Adenocarcinoma, Saudi Arabia), THCA-US (Thyroid Adenocarcinoma, USA), ORCA-IN (Oral Cancer, India), KIRC-US (Kidney Renal Clear Cell Carcinoma, USA), and LAML-KR (Acute Myeloid Leukemia, Korea).

References

D'Hont, A., et al. (2012). The banana (musa acuminata) genome and the evolution of monocotyledonous plants. *Nature*, **488**(7410), 213–217.

Xu, F., et al. (2012). A fast and accurate SNP detection algorithm for next-generation sequencing data. *Nat. Commun.*, **3**, 1258.

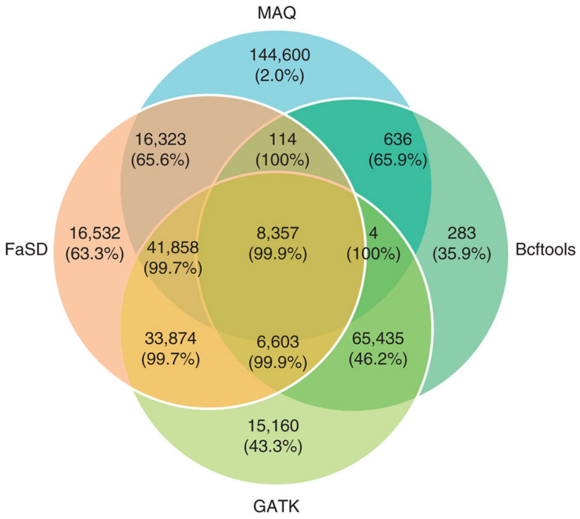

(a)

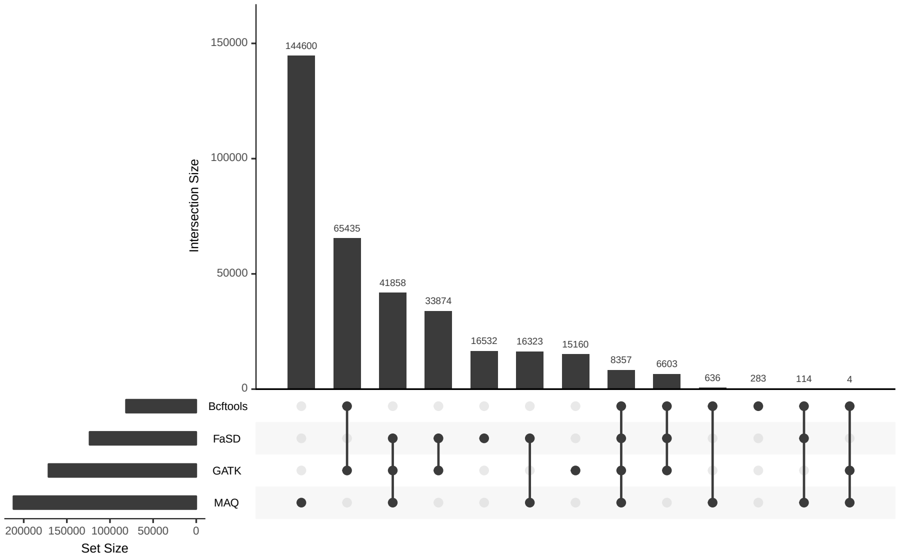

(b)

Supplementary Figure 1: *Intersections of variants called by different tools. (a) Euler diagram with four sets showing overlapping SNP calls obtained from four different variant calling tools (MAQ, Bcftools, GATK, FaSD) on NGS data. The percentage under each count is fraction of SNPs confirmed by the Affymetrix SNP array. Figure from Xu, F., et al. (2012) with permission of the publisher. (b) UpSetR version of the Euler diagram shown in (a).*

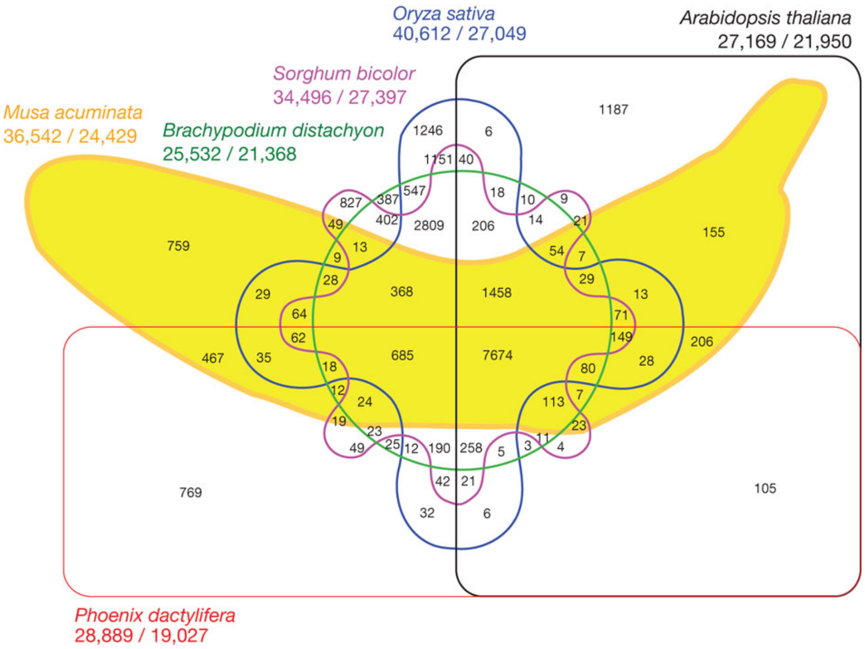

(a)

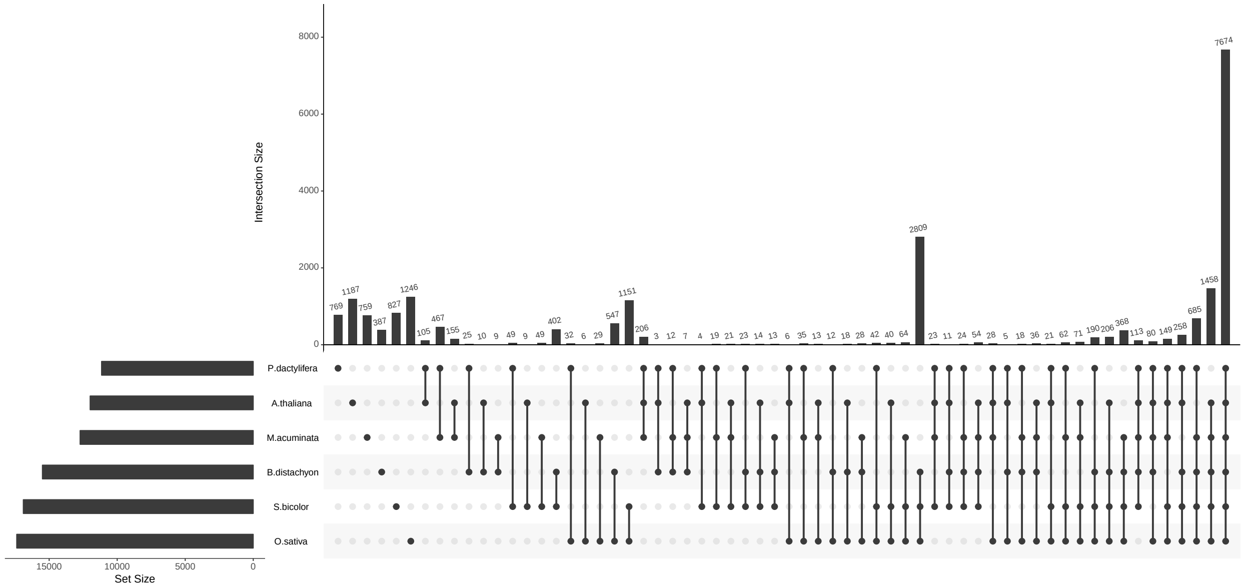

(b)

Supplementary Figure 2: *Shared genes of plant species. (a) Venn diagram with 6 sets showing intersection of gene clusters between the banana (Musa acuminata) genome and the genome of five other plant species. Figure taken from D'Hont, A., et al. (2012) with permission of the publisher. (b) UpSetR version of the Venn diagram in (a), ordered by cardinality.*

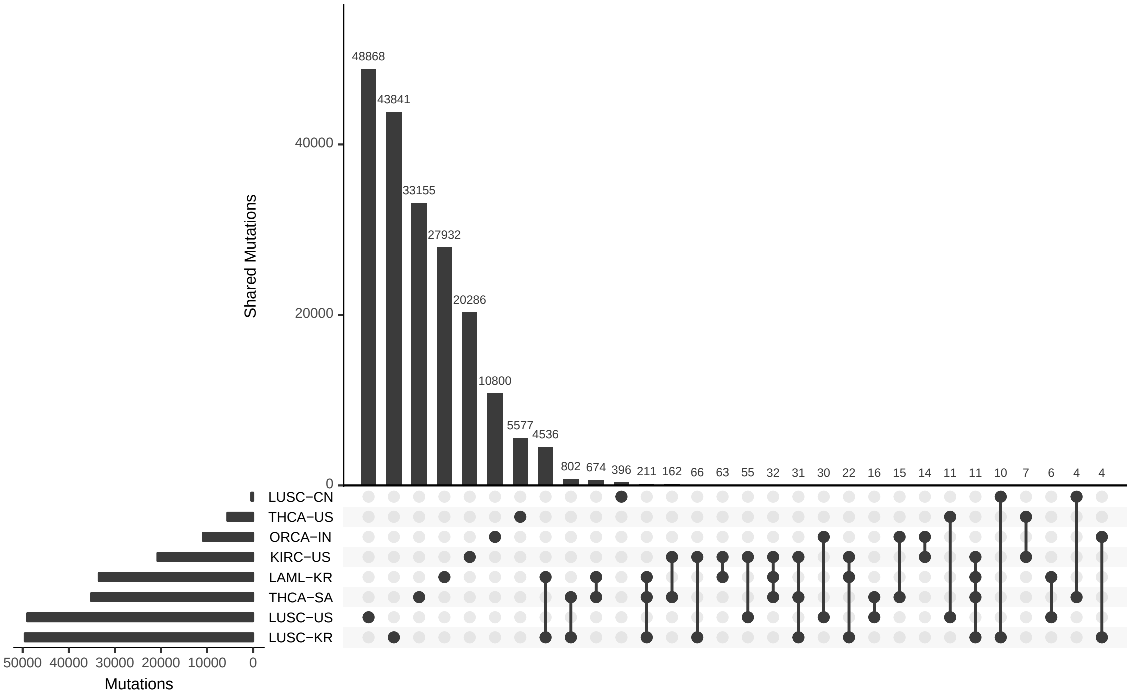

Supplementary Figure 3: *An UpSetR plot of the ICGC data. Non-empty intersections showing overlap of variants across selected cohorts.*

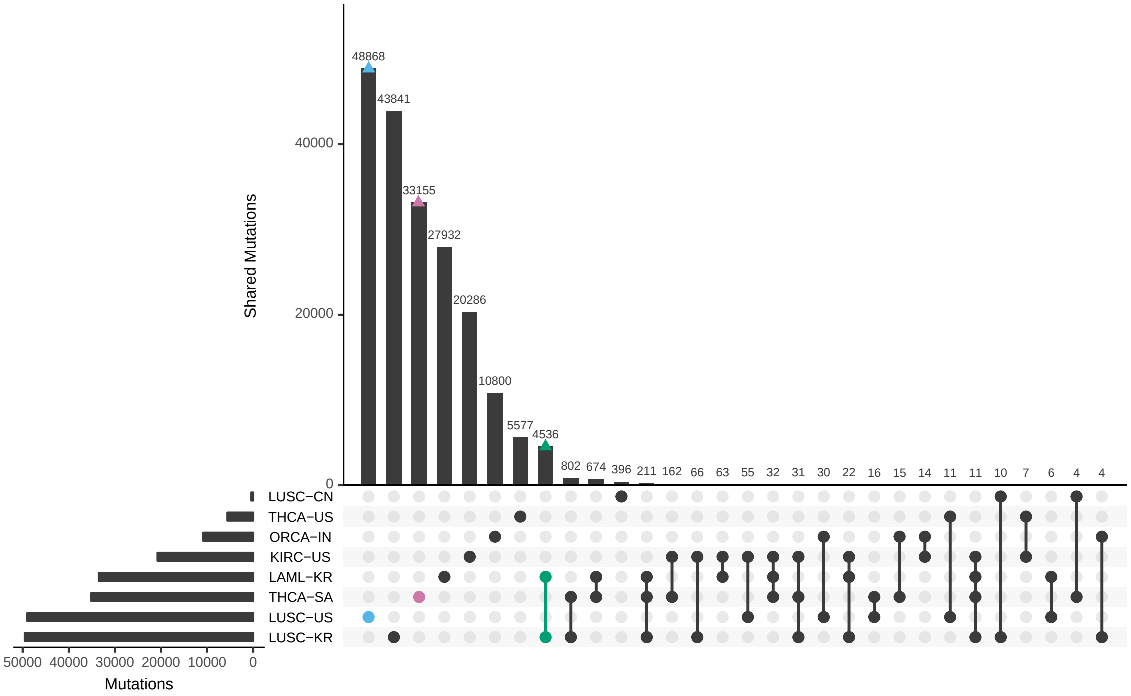

Supplementary Figure 4: *Intersection queries on unique and shared variants across cancer studies. The intersection queries shown are the one-way intersections of LUSC-US (blue) and THCA-SA (purple), and the two-way intersection of LUSC-KR and LAML-KR (green). The intersection queries shown are not set to "active", hence a triangular tick is displayed as opposed to an overlapping bar (see Supplementary Figure 5).*

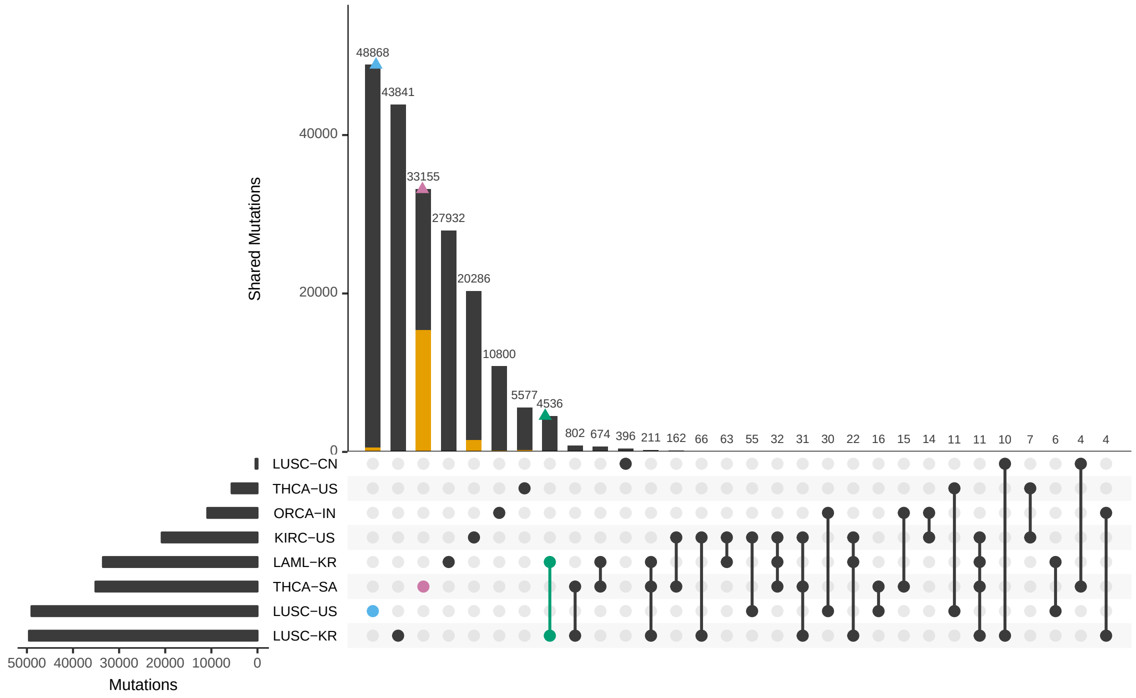

Supplementary Figure 5: *An UpSetR plot containing the three intersection queries introduced in Supplementary Figure 4, and an element query on variants classified as deletions. Since the newly added query is an element query, it displays the distribution of elements within the query across all of the intersections shown. The element query is set to "active" which overlays bars on top of the intersection size bar chart instead of using triangular ticks.*
